# Open Chromatin Profiling in Adipose Tissue Marks Genomic Regions with Functional Roles in Cardiometabolic Traits

**DOI:** 10.1534/g3.119.400294

**Published:** 2019-06-11

**Authors:** Maren E. Cannon, Kevin W. Currin, Kristin L. Young, Hannah J. Perrin, Swarooparani Vadlamudi, Alexias Safi, Lingyun Song, Ying Wu, Martin Wabitsch, Markku Laakso, Gregory E. Crawford, Karen L. Mohlke

**Affiliations:** * Department of Genetics,; † Department of Epidemiology, University of North Carolina at Chapel Hill, Chapel Hill, NC 27599,; ‡ Department of Pediatrics, Division of Medical Genetics and Center for Genomic and Computational Biology, Duke University, NC 27710,; § Department of Pediatrics and Adolescents Medicine, Division of Pediatric Endocrinology and Diabetes, University of Ulm, Germany 89081, and; ** Department of Medicine, University of Eastern Finland and Kuopio University Hospital, Kuopio, Finland 70210

**Keywords:** chromatin accessibility, adipose tissue, preadipocytes, GWAS, cardiometabolic trait

## Abstract

Identifying the regulatory mechanisms of genome-wide association study (GWAS) loci affecting adipose tissue has been restricted due to limited characterization of adipose transcriptional regulatory elements. We profiled chromatin accessibility in three frozen human subcutaneous adipose tissue needle biopsies and preadipocytes and adipocytes from the Simpson Golabi-Behmel Syndrome (SGBS) cell strain using an assay for transposase-accessible chromatin (ATAC-seq). We identified 68,571 representative accessible chromatin regions (peaks) across adipose tissue samples (FDR < 5%). GWAS loci for eight cardiometabolic traits were enriched in these peaks (*P* < 0.005), with the strongest enrichment for waist-hip ratio. Of 110 recently described cardiometabolic GWAS loci colocalized with adipose tissue eQTLs, 59 loci had one or more variants overlapping an adipose tissue peak. Annotated variants at the *SNX10* waist-hip ratio locus and the *ATP2A1-SH2B1* body mass index locus showed allelic differences in regulatory assays. These adipose tissue accessible chromatin regions elucidate genetic variants that may alter adipose tissue function to impact cardiometabolic traits.

Dysregulation of genes expressed in adipose tissue influences cardiometabolic traits and diseases. Subcutaneous adipose tissue serves as a buffering system for lipid energy balance, particularly fatty acids,(Coelho *et al.* 2013; Fernández-Veledo *et al.* 2009; Gustafson *et al.* 2015) and may play a protective role in cardiometabolic risk.(Porter *et al.* 2009) Subcutaneous adipose expression quantitative trait loci (eQTL) studies have identified genes involved in central obesity and metabolic traits,(Emilsson *et al.* 2008; Zhong *et al.* 2010; Greenawalt *et al.* 2011; Nica *et al.* 2011; Civelek *et al.* 2017) and specific cardiometabolic genome-wide association study (GWAS) loci have been shown to colocalize with subcutaneous adipose eQTLs.(Global Lipids Genetics Consortium 2013; DIAbetes Genetics Replication and Meta-analysis (DIAGRAM) Consortium *et al.* 2014; Shungin *et al.* 2015; Locke *et al.* 2015; Civelek *et al.* 2017) In addition, a recent GWAS study of waist-hip ratio, a measure of central obesity, identified loci that were enriched both for putative regulatory elements in adipose nuclei and for genes expressed in subcutaneous adipose tissue,(Shungin *et al.* 2015) many of which have been linked to adipose function.(Dahlman *et al.* 2016) Identification and characterization of adipose tissue regulatory regions and variants would improve understanding of biological processes and the mechanisms underlying cardiometabolic loci.

Adipose tissue is composed of many cell types, including adipocytes, preadipocytes, vascular cells, immune cells, and nerve cells.(Lynes and Tseng 2018) Characterization of heterogeneous whole adipose tissue and its component cell types are both needed to fully delineate the role of adipose tissue in cardiometabolic disease. Human adipose tissue samples can be used to identify differences in chromatin accessibility due to genotype and link variants to cardiometabolic traits; however, samples may also differ due to site of tissue extraction, sample handling and storage conditions, and environmental contributions. Although cell models do not fully replicate cells within a complex tissue, their growth, storage, and environmental conditions can be controlled. Cells from the Simpson Golabi-Behmel Syndrome (SGBS) human preadipocyte cell strain are diploid, easy to grow in culture, can be differentiated to mature adipocytes(Fischer-Posovszky *et al.* 2008) and are exposed to less experimental variation than primary human preadipocytes due to genotype or sample collection differences.

Adipose tissue and adipocytes are poorly represented in chromatin accessibility datasets because the high lipid content makes experimental assays challenging. To date, for human adipose tissue or adipocytes, only three DNase-seq datasets(Loft *et al.* 2015; Schmidt *et al.* 2015b) and three ATAC-seq datasets(Allum *et al.* 2015; ENCODE Consortium 2012) are available. In addition to chromatin accessibility, chromatin immunoprecipitation (ChIP)-seq for histone marks have been characterized in adipose nuclei from subcutaneous adipose tissue and in differentiated adipocytes from mesenchymal stem cells (Roadmap Epigenomics Project), and these data were integrated to annotate genomic regions into chromatin states characteristic of regulatory functions such as promoters, enhancers, or insulators.(Roadmap Epigenomics Consortium 2015) Regions of chromatin accessibility in many cell types are located preferentially in regulatory regions,(Roadmap Epigenomics Consortium 2015; Scott *et al.* 2016) suggesting that chromatin accessibility maps can improve accuracy of predicting regulatory chromatin states in adipose cell types.

Chromatin accessibility data can be used to characterize candidate variants at noncoding GWAS loci. Allelic differences have been found in levels of accessible chromatin, transcription factor binding, and histone marks of chromatin state,(Degner *et al.* 2012; Kasowski *et al.* 2013; Kilpinen *et al.* 2013; McVicker *et al.* 2013; Leung *et al.* 2015; Kumasaka *et al.* 2016) and these differences have provided a functional context for interpreting GWAS loci.(Gate *et al.* 2018; Astle *et al.* 2016; Roman *et al.* 2015) Identifying transcription factor motifs and footprints in accessible chromatin regions can be used to predict transcription factor binding sites.(Varshney *et al.* 2017) Improved annotation of candidate regulatory variants and candidate transcription factors in adipose tissue could aid identification of molecular mechanisms at GWAS loci.

In this study, we performed ATAC-seq on frozen clinical subcutaneous adipose tissue needle biopsy samples and SGBS preadipocytes and adipocytes to identify regions of accessible chromatin for each sample type. We identified cardiometabolic GWAS loci and transcription factor binding motifs in ATAC-seq open chromatin regions and used the ATAC-seq annotations to characterize candidate variants at cardiometabolic GWAS loci with colocalized adipose tissue eQTL associations. Finally, through experimental analysis of allelic differences in regulatory functions, we report functional non-coding variants at two cardiometabolic GWAS loci.

## Materials and Methods

### METSIM study participants

Subcutaneous adipose tissue needle biopsies were obtained from METabolic Syndrome in Men (METSIM) participants as previously described.(Civelek *et al.* 2017) We used three adipose tissue needle biopsy samples for ATAC-seq (Table S1). The METSIM study includes 10,197 men, aged from 45 to 73 years, randomly selected from Kuopio, Eastern Finland, and examined in 2005 – 2010.(Stancakova *et al.* 2009; Laakso *et al.* 2017) The Ethics Committee of the University of Eastern Finland in Kuopio and the Kuopio University Hospital approved the METSIM study and it was carried out in accordance with the Helsinki Declaration. DNA samples were genotyped on the Illumina OmniExpress and HumanCoreExome arrays and imputed using the Haplotype Reference Consortium(McCarthy *et al.* 2016) as previously described.(Civelek *et al.* 2017)

### Sample processing and ATAC-seq library preparation

Human adipose tissue was flash frozen and stored at -80° until use. For adipose tissue samples 1 and 3, we generated libraries using nuclei isolation buffers that contained detergent (1% NP-40) or did not contain detergent. For tissue sample 2, we generated libraries using ∼12 mg or ∼36 mg of tissue and contained detergent. Replicates including detergent and less tissue in library preparation resulted in a greater number of peaks and higher peak similarity between individuals compared to no detergent (Table S11). From these observations, we performed all subsequent analyses with the three detergent-treated replicates. Tissue was pulverized in liquid nitrogen using a Cell Crusher homogenizer (cellcrusher.com). The tissue powder was resuspended in nuclei isolation buffer (20 mM Tris-HCl, 50 mM EDTA, 60 mM KCl, 40% glycerol, 5 mM spermidine, 0.15 mM spermine, 0.1% mercaptoethanol, 1% NP-40). Tubes were rotated at 4° for 5 min. The solution was homogenized using a tight homogenizer (Wheaton) for 10 strokes and was centrifuged at 1500 × g for 10 min at 4°. Following removal of the lipid layer and supernatant, the pellet was resuspended in buffer (10 mM Tris-HCl, 10 mM NaCl, 3 mM MgCl_2_) and centrifuged at 1200 × g for 10 min at 4°. The supernatant was removed and the pellet was used for the transposase reaction as previously described.(Buenrostro *et al.* 2013) We used 2.5 ul Tn5 for adipose tissue libraries. Following library PCR amplification for adipose tissue, we removed primer dimers using Ampure Beads (Agencourt) with a 1:1.2 ratio of library to beads. Libraries were visualized and quantified using a TapeStation or Bioanalyzer and sequenced with 50-bp reads on an Illumina Hi-Seq 2500 at the Duke University Genome Sequencing shared resource facility (single-end sequencing).

SGBS cells(Wabitsch *et al.* 2001) were generously provided by Dr. Martin Wabitsch (University of Ulm) and cultured as previously described.(Cannon *et al.* 2017) To differentiate SGBS cells, SGBS preadipocytes were cultured in serum-containing medium until confluent, then rinsed in PBS and differentiated for four days in basal medium (DMEM:F12 + 3.3mM biotin + 1.7mM panthotenate) supplemented with 0.01 mg/mL transferrin, 20 nM insulin, 200 nM cortisol, 0.4 nM triiodothyronine, 50 nM dexamethasone, 500 uM IBMX, and 2 uM rosiglitazone. After four days, differentiated SGBS cells were maintained in basal medium supplemented with 0.01 mg/mL transferrin, 20 nM insulin, 200 nM cortisol, 0.4 nM triiodothyronine. We generated profiles with 50,000 cells following the Omni-ATAC protocol(Corces *et al.* 2017) (Table S11). We removed primer dimers using Zymo DNA Clean and Concentrator, visualized and quantified libraries using a TapeStation or Bioanalyzer, and sequenced with 50-bp reads on an Illumina Hi-Seq 4000 at the University of North Carolina High-Throughput Sequencing Facility (paired-end sequencing).

### ATAC-seq alignment and peak calling

We obtained previously published adipose ATAC-seq datasets from subcutaneous adipose tissue (ENCODE ENCSR540BML),(ENCODE Consortium 2012) tissue-derived adipocytes,(Allum *et al.* 2015) and GM12878 lymphoblasts.(Buenrostro *et al.* 2013) The tissue-derived adipocyte ATAC-seq data were shared by the McGill Epigenomics Mapping Centre and is available from the European Genome-phenome Archive of the European Bioinformatics Institute (dataset EGAD00001001300).

To minimize mapping differences between read length and single-end *vs.* paired-end samples, we merged the mate pair fastq files and trimmed reads to 50 nucleotides for each paired-end ATAC-seq sample and aligned reads from all samples as single-end. We removed sequencing adapters from raw ATAC-seq sequence reads using Tagdust (Lassmann *et al.* 2009) with a false discovery rate of 0.1% and selected high quality reads with a Phred score of at least 20 for at least 90% of bases using the FASTX toolkit (http://hannonlab.cshl.edu/fastx_toolkit). We aligned filtered reads to the hg19 human genome using bowtie2(Langmead and Salzberg 2012), penalizing ambiguous bases as mismatches. We removed any alignments with mapping quality less than 20, mitochondrial reads, or blacklisted regions (Quinlan 2014; Karolchik *et al.* 2004) and shifted the resulting alignments by +4 on the + strand and -5 on the – strand so that the 5′ base of each alignment corresponded to the center of the binding site of the Tn5 transposase(Adey *et al.* 2010; Buenrostro *et al.* 2013). For the METSIM adipose tissue samples, we verified sample identity using verifyBamID (Jun *et al.* 2012) using genotyped variants with at least 10 ATAC-seq reads in the sample with the lowest read depth (Tissue 2; 8,683 variants), minimum minor allele frequency of 0.01, and call rate of at least 0.5; we used the best-matched genotypes for each sample. For all samples, we called peaks using MACS2(Zhang *et al.* 2008) with no background dataset, smoothing ATAC-seq signal over a 200 bp window centered on the Tn5 integration site, allowing no duplicates, and a false discovery rate (FDR)<5%; we refer to peaks called on reads from technical replicate samples (SGBS adipocytes, SGBS preadipocytes, tissue-derived adipocytes, and GM12878 lymphoblasts) as ‘replicate peaks’.

### Representative ATAC-seq peaks

For samples with technical replicates, we pooled reads across replicates and called peaks (MACS2, FDR < 5%), and then defined the portion of these peaks that shared at least one base with a replicate peak in two or more replicates as ‘representative peaks’. The METSIM adipose tissue samples are from different individuals and are not technical replicates. Due to a low number of samples, we used the union of peaks across individuals as representative peaks. Unless otherwise noted, we selected the top 50,000 representative peaks in each group for downstream analyses. For the groups with technical replicates and the single ENCODE adipose tissue sample, we selected the top 50,000 representative peaks with the most significant peak p-values. For METSIM adipose tissue, we ranked the peak p-values in each individual (with 1 being the strongest) and used the average of these ranks to select the top 50,000 representative peaks. This approach reduced the chance that outlier p-values from a single individual would bias peak rank.

### ATAC-seq principal component analysis

We generated a total set of accessible chromatin regions by taking the top 50,000 peaks in each group of ATAC-seq samples. For each ATAC-seq sample, we counted the number of non-duplicated nuclear reads overlapping the total set of accessible chromatin regions using featureCounts.(Liao *et al.* 2014) We performed library size normalization and variance stabilization using the regularized log (rlog) function in DESeq2.(Love *et al.* 2014) We performed principal component analysis (PCA) using a modified version of the DESeq2 plotPCA function.

### Peak genomic distribution and overlap with Roadmap chromatin states

We determined the location of ATAC-seq peaks relative to genes from the GENCODE 24lift37 Basic Set. Using BEDTools,(Quinlan 2014) we divided peaks into the following categories: TSS-proximal (5 kb upstream to 1 kb downstream of a GENCODE transcription start site), intragenic (within a gene body but not within TSS-proximal regions), downstream (within 5 kb downstream of a transcription termination site but not within any gene body), and distal (>5 kb from either end of any gene). We obtained chromatin states for an 18-state model based on ChIP-seq data for 98 cell and tissue types using 6 histone marks (H3K4me1, H3K4me3, H3K36me3, H3K27me3, H3K9me3, and H3K27ac) from the Roadmap Epigenomics Consortium.(Roadmap Epigenomics Consortium 2015) We generated the following combined states by merging states of similar genomic context: promoter (1_TssA, 2_TssFlnk, 3_TssFlnkU, 4_TssFlnkD, 14_TssBiv), transcribed (5_Tx, 6_TxWk), enhancer (7_EnhG1, 8_EnhG2, 9_EnhA1, 10_EnhA2, 11_EnhWk, 15_EnhBiv), and polycomb repressed (16_ReprPC, 17_ReprPCWk). Using BEDTools(Quinlan 2014) we calculated the number of representative ATAC-seq peak bases that overlapped each chromatin state. We ranked the ATAC-seq peak overlap of each chromatin state in adipose nuclei (Roadmap epigenome ID E063) relative to all other cell types, where a rank of 1 corresponds to largest amount of overlap compared to all other cell types.

### Enrichment of transcription factor motifs within ATAC-seq peaks

We tested for enrichment of 519 transcription factor binding motifs from the JASPAR core 2016 vertebrates database(Mathelier *et al.* 2016) within the top 50,000 representative peaks for adipose tissue and GM12878 lymphoblasts using Analysis of Motif Enrichment (AME)(McLeay and Bailey 2010). We used shuffled peak sequences with preserved dinucleotide content as background for the enrichment and the Fisher Exact Test to calculate enrichment significance. We classified motifs with an Expect value (E) less than 1x10^−100^ as significantly enriched.

### Transcription factor motif scanning and footprinting within ATAC-seq peaks

To identify transcription factor motifs both disrupted and generated by GWAS variants, we constructed personalized reference genomes (hg19) with the –create_reference option in the AA-ALIGNER pipeline(Buchkovich *et al.* 2015) using genotypes in the adipose tissue samples. We scanned the resulting haplotypes for 519 transcription factor binding motifs from the JASPAR core 2016 vertebrates database using FIMO.(Mathelier *et al.* 2016; Grant *et al.* 2011) If two motifs for the same factor existed at the exact same genomic coordinates and on the same strand on each haplotype, we used the motif with the highest motif score.

We performed transcription factor footprinting for 35 transcription factor motifs corresponding to 34 unique adipose-related transcription factors (Table S8). The 34 transcription factors included 21 described as adipose core transcription factors(Saint-André *et al.* 2016), six dimer motifs that contained a core transcription factor, plus CEBPA, CEBPB, CEBPD, ZEB1, SPI1, SPIB, and CT*CF*. For the resulting motifs, we generated windows containing the genomic coordinates of the motif and 100 bp flanking both motif edges. We removed motif windows where fewer than 90% of bases could be uniquely mapped or that overlapped blacklisted regions.(Karolchik *et al.* 2004; Li *et al.* 2009; Quinlan 2014) We constructed matrices of the number of Tn5 transpositions across the remaining motif windows and predicted which motifs were likely bound using CENTIPEDE.(Pique-Regi *et al.* 2011) We used motif scores calculated by FIMO for CENTIPEDE priors and classified a motif with a CENTIPEDE posterior binding probability greater than 0.99 as bound and less than 0.5 as unbound.

Next, we determined which transcription factors exhibited an average decrease in ATAC-seq signal across their motifs relative to flanking regions, termed an aggregate footprint profile; we considered these footprints to be the most robust and consistent footprints across all motif sites. We calculated the average transposition probability at each window position separately for bound and the top 10,000 unbound sites to obtain aggregate bound and unbound profiles, calculated the transposition probability ratio (TPR) by dividing each position in the bound profiles by the corresponding position in the unbound profiles, and then calculated the average TPR across the motifs (mTPR) and the 100 bp flanking regions (fTPR). We considered transcription factor motifs to display an aggregate footprint profile if mTPR was less than fTPR.

### Enrichment of GWAS variants in ATAC-seq peaks

We tested for enrichment of genetic variants in ATAC-seq peaks using GREGOR, which compares overlap of GWAS variants relative to control variants matched for number of LD proxies, allele frequency, and gene proximity.(Schmidt *et al.* 2015a) We selected lead variants with a p-value less than 5×10^−8^ from 11 trait categories from the GWAS catalog (December 2016): type 2 diabetes, insulin, glucose, cardiovascular outcomes, blood pressure traits, low-density lipoprotein cholesterol (LDL-C), high-density lipoprotein cholesterol (HDL-C), triglycerides, total cholesterol, body mass index (BMI), and waist-hip ratio adjusted for BMI (WHR). Phenotypes included in trait categories are listed in Table S5. Loci that were associated with multiple traits were assigned to each trait. To remove multiple lead variants for the same association signal, we performed LD clumping using swiss (https://github.com/welchr/swiss) with the 1000G_2014-11_EUR LD reference; variants in moderate LD (*r^2^* > 0.2) and within 1 Mb of a variant with a more significant p-value were removed. We used GREGOR to test for enrichment of the resulting GWAS lead variants or their LD proxies (*r^2^* threshold of 0.8 within 1 Mb of the GWAS lead, 1000 Genomes Phase I) in ATAC-seq peaks relative to control variants. We tested for enrichment in the top 50,000 representative peaks for adipose tissue, SGBS adipocytes, SGBS preadipocytes, and GM12878 lymphoblasts. Enrichment was considered significant if the enrichment p-value was less than the Bonferroni-corrected threshold of 5×10^−3^ (0.05/11 trait groups). To compare enrichment magnitudes between regions and traits, we calculated an enrichment z-score:

$$z-score=\frac{\text{observed}\;\text{overlaps}-\text{expected}\;\text{overlaps}}{\text{standard}\;\text{deviation}}$$

The expected overlaps and standard deviation were estimated using GREGOR.(Schmidt *et al.* 2015a) We visualized the enrichment results using the heatmap.2 function in the gplots R package.(R Core Team 2016; Warnes *et al.* 2016).

### Overlap of GWAS-eQTL colocalized loci with ATAC-seq peaks

eQTL mapping in 770 subcutaneous adipose tissue samples and determination of GWAS-coincident eQTLs was described previously. (Civelek *et al.* 2017; Cannon *et al.* 2017) We identified overlap of ATAC-seq peaks with any variant in LD (*r^2^* > 0.8) with the GWAS lead variant at 110 loci (6,692 variants) using BEDTools.(Quinlan 2014) LD was calculated using the 770 METSIM individuals included in the eQTL analysis.

### Transcriptional reporter luciferase assays

SGBS preadipocyte, 3T3-L1 preadipocyte, SW872 liposarcoma, and THP-1 monocyte cells were maintained and transcriptional reporter luciferase assays were performed as previously described.(Cannon *et al.* 2017; Schick *et al.* 2016) 3T3-L1 preadipocytes (ATCC, CL-173) were differentiated as described in the ATCC protocol. Table S12 contains primers used for amplifying ATAC-seq peaks overlapping the variant of interest. Amplified regions were inserted in pGL4.23 firefly luciferase reporter vectors (Promega) upstream of the minimal promoter and luciferase gene. We cloned two sizes of constructs for rs7187776 due to a restriction enzyme site in the middle of the larger construct; we tested both in luciferase assays. The long construct includes part of the 3′ UTR of *TUFM* and part of the 5′ UTR of *SH2B1*. Fragments containing potential enhancers are designated as ‘forward’ or ‘reverse’ based on their orientation with respect to the genome. Regions were designed to include the entire ATAC-seq peak overlapping the variant of interest. Three to five independent clones were cotransfected with *Renilla* luciferase vector in triplicate (SGBS, 3T3-L1 adipocytes) or duplicate (SW872, THP1, 3T3-L1 preadipocytes) wells using Lipofectamine 3000 (SGBS, THP-1, Life Technologies), Lipofectamine 2000 (3T3-L1 preadipocytes and adipocytes) or FUGENE 6 (SW872, Promega). Firefly luciferase activity of the clones containing the PCR fragments was normalized to *Renilla* luciferase readings to control for differences in transfection efficiency. We repeated all luciferase transcriptional reporter experiments on independent days and obtained consistent results. Data are reported as fold change in activity relative to an empty pGL4.23 vector. We used two-sided Student’s *t*-tests to compare luciferase activity.

### Electrophoretic mobility shift assays (EMSA)

For EMSA, we prepared nuclear cell extracts from SGBS preadipocyte and SW872 cells using the NE-PER nuclear and cytoplasmic extraction kit (Thermo Scientific) as previously described.(Kulzer *et al.* 2014) Double-stranded oligos (Table S12) were incubated with SGBS preadipocyte or SW872 nuclear extract or 100 ng purified PU.1 protein (Creative Biomart SPI1-172H) and DNA-protein complex visualization was carried out as previously described.(Kulzer *et al.* 2014) A positive control oligo contained the PU.1 motif from JASPAR and a negative control did not contain the motif (Table S12). We repeated all EMSA experiments on independent days and obtained consistent results.

### Allelic imbalance

We aligned reads for the adipose tissue samples to personalized genomes using the allele-aware aligner GSNAP allowing two mismatches, no indels, and treating ambiguous bases (encoded as N’s) as mismatches.(Wu and Nacu 2010) We extracted unique alignments and filtered alignments to the mitochondrial genome and blacklisted regions.(Li *et al.* 2009; Karolchik *et al.* 2004) Using WASP,(van de Geijn *et al.* 2015) we removed alignments that did not uniquely map to each allele at heterozygous sites. Allele count pileup files were generated at heterozygous sites with a minimum base quality Phred score of 30 to minimize the impact of sequencing errors using samtools. We removed heterozygous loci with aligned bases other than the two genotyped alleles and selected heterozygous sites with at least 10 total counts and at least 1 count per allele. To account for residual biases, we fit allele counts to a beta-binomial distribution with the probability of success (reference allele ratio) and dispersion estimated using maximum likelihood separately for each sample using the VGAM R package.(Yee 1996; R Core Team 2016) We performed two-tailed beta-binomial tests of allelic imbalance using VGAM.

To confirm allelic imbalance in PU.1 binding and chromatin accessibility at rs7187776 (genomic position chr16:28857645), we analyzed public genotype, SPI1 ChIP-seq, and DNase-seq data for the GM12891 cell line. We obtained genotypes for individual NA12891 from ftp://ftp-trace.ncbi.nih.gov/1000genomes/. We downloaded GM12891 SPI1 ChIP-seq alignments (ENCFF450BQJ, ENCFF152ZGE) and DNase-seq alignments (ENCFF070BAN) from ENCODE. Allele count pileup files were generated at heterozygous sites with a minimum base quality Phred score of 30 to minimize the impact of sequencing errors using samtools.

### Data availability

ATAC-seq reads (SGBS preadipocytes and adipocytes) and peaks (adipose tissue, SGBS preadipocytes and adipocytes) can be accessed from GEO: accession number GSE110734.

Supplementary tables and figures are available at FigShare: https://doi.org/10.25387/g3.8120933.

## Results

### Chromatin accessibility in frozen adipose tissue and SGBS preadipocytes and adipocytes

We generated ATAC-seq open chromatin profiles from three frozen subcutaneous adipose tissue needle biopsy samples (Table S1), two replicates of SGBS preadipocytes, and three replicates of SGBS adipocytes. In the adipose tissue samples, we generated ∼56-70 million non-duplicated nuclear reads and ∼36-58 thousand peaks (FDR < 5%, Table 1, Methods). We identified 68,571 representative adipose tissue peaks by taking the union of peaks across the three samples. We generated a comparable number of non-duplicated nuclear reads in the SGBS samples (∼30-90 million), but identified many more peaks (122,924 and 164,252 representative peaks for SGBS preadipocytes and adipocytes respectively) (Table 1, Methods). The lower signal-to-noise of adipose tissue profiles compared to cultured, largely homogeneous SGBS cells is expected due to the heterogeneity of whole adipose tissue and stress resulting from sample freezing.

**Table 1:** ATAC-seq alignment metrics of human adipose tissue and SGBS preadipocytes and adipocytes

Sample	Total reads	Aligned reads	Percent mitochondrial reads	Nuclear alignments	Remaining reads after blacklist filtering	Remaining reads after duplicates removed	Number of peaks*^b^*
Tissue 1	129.5	87.4	8.5	80.0	79.0	70.6	58,550
Tissue 2	131.5	83.6	12.8	72.9	71.8	60.6	36,785
Tissue 3	119.3	70.5	11.9	62.2	61.3	57.1	49,962
SGBS adipocytes 1*^a^*	382.6	275.9	2.1	268.6	267.7	90.4	184,455
SGBS adipocytes 2*^a^*	245.1	172.9	1.9	168.7	168.1	84.1	172,247
SGBS adipocytes 3*^a^*	253.7	181.0	1.5	177.2	176.7	87.5	191,141
SGBS preadipocytes 1*^a^*	97.3	71.8	1.0	70.8	70.7	34.6	171,279
SGBS preadipocytes 2*^a^*	75.1	54.1	1.1	53.3	53.1	30.5	139,911

Reads are reported in millions of reads.

a Samples were sequenced using paired-end reads, but processed as single-end reads.

b We identified 68,571 representative peaks across adipose tissue, 122,924 across SGBS preadipocytes, and 164,252 across SGBS adipocyte samples.

**Figure 1:**
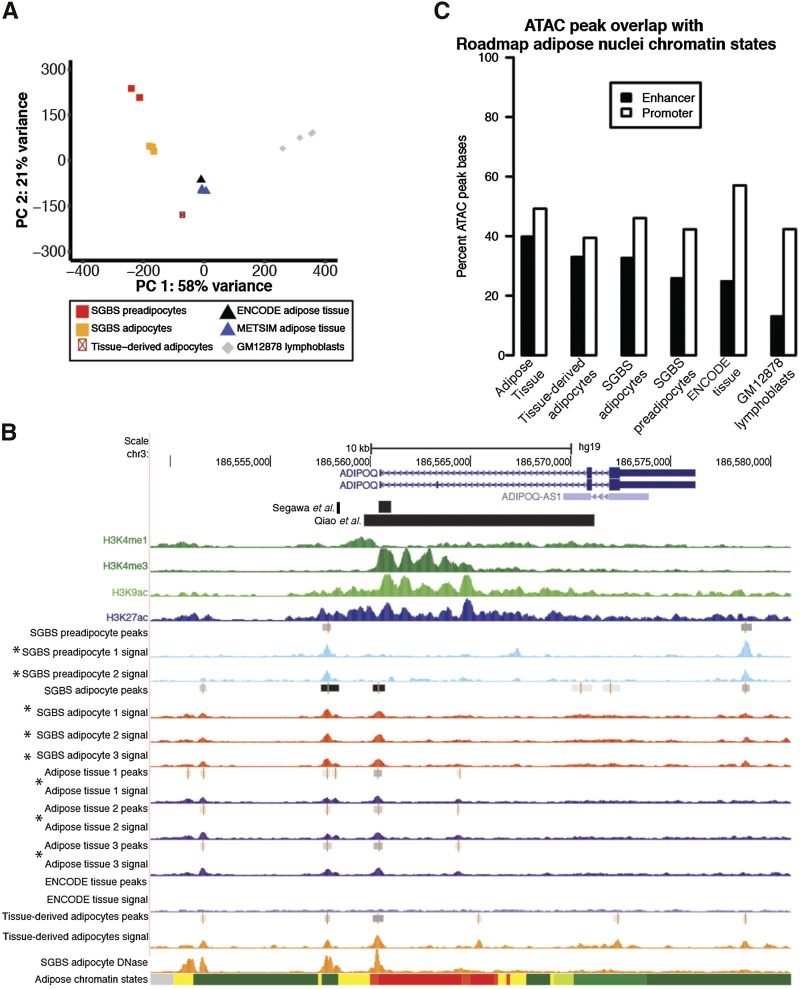
Comparison of ATAC-seq read profiles and peaks between samples and with Roadmap adipose nuclei chromatin states. (A) Principal component analysis (PCA) of ATAC-seq read counts within representative peaks. (B) UCSC genome browser image (hg19) showing the *ADIPOQ* gene regions. ChIP-seq for histone marks from the Roadmap Epigenomics project adipose nuclei are shown at the top in green and blue. ATAC-seq signal tracks are shown in different colors by source: SGBS preadipocytes in light blue, SGBS adipocytes in red, adipose tissue in purple, ENCODE adipose tissue in light purple, and tissue-derived adipocytes in orange. DNase hypersensitivity signal tracks for SGBS adipocytes are also shown in orange. Asterisks represent ATAC-seq data generated in this manuscript. Peak regions are indicated by gray bars. The bottom track shows chromatin states from the Roadmap Epigenomics Project for adipose nuclei (yellow = enhancer; green = transcribed; orange/red = promoter; light green = genic enhancer; gray = repressed/polycomb; light red = bivalent/poised TSS; turquoise = heterochromatin). (C) Overlap of the top 50,000 ATAC-seq peaks with promoter and enhancer chromatin states identified in Roadmap adipose nuclei.

Using principal component analysis of ATAC-seq read counts within representative peaks, we identified that adipose tissue, SGBS preadipocyte, and SGBS adipocyte samples cluster into three distinct groups with strong within-group similarity (Figure 1A). The adipose tissue profiles were more similar to SGBS adipocyte profiles than to SGBS preadipocyte profiles (Figure 1A), suggesting the adipose tissue samples contain more adipocytes than preadipocytes.

We tested for enrichment of 519 transcription factor binding motifs from the JASPAR database in the top 50,000 representative adipose tissue ATAC-seq peaks using AME.(Mathelier *et al.* 2016; McLeay and Bailey 2010) We identified 162 significantly enriched motifs (E < 1x10^−100^), including 41 motifs enriched in adipose tissue but not lymphoblasts (Table S2). The set of 41 contains motifs for transcription factors known to promote adipogenesis, such as CEBP family members, STAT family members, and PPARG.(Sarjeant and Stephens 2012)

To evaluate the distribution of ATAC-seq peaks across samples, we examined the accessible chromatin landscape at *ADIPOQ*, which encodes adiponectin, a hormone secreted by adipocytes that is not expressed in preadipocytes.(Ambele *et al.* 2016; Körner *et al.* 2005) Adipose tissue and SGBS adipocyte ATAC-seq peaks overlapped the transcription start site (TSS) and parts of previously described regulatory elements upstream and in intron 1 of *ADIPOQ* that showed increased transcriptional activity in reporter assays(Segawa *et al.* 2009; Qiao *et al.* 2005) (Figure 1B). Additionally, a strong ATAC-seq peak downstream of *ADIPOQ* was present in SGBS preadipocytes, suggesting this region may harbor preadipocyte-specific regulatory elements. These data demonstrate that reproducible ATAC-seq open chromatin profiles can be obtained from small amounts (12-36 mg, one-third to two-thirds of a needle biopsy) of frozen clinical subcutaneous adipose tissue samples and SGBS preadipocytes and adipocytes.

### Comparison of adipose tissue, adipocyte, and preadipocyte open chromatin

We compared our adipose tissue and SGBS representative ATAC-seq peaks to existing ATAC-seq datasets from tissue-derived adipocytes,(Allum *et al.* 2015) ENCODE subcutaneous adipose tissue, and GM12878 lymphoblasts (outgroup) using three methods. First, principal component analysis of read counts within representative peaks shows that our adipose tissue profiles were most similar to ENCODE adipose tissue and tissue-derived adipocyte profiles (Figure 1A). These tissue-derived adipocyte and ENCODE adipose tissue profiles were also more similar to SGBS adipocytes than SGBS preadipocytes. Our adipose tissue and SGBS profiles were more similar to existing adipocyte profiles than to GM12878 profiles.

Second, we compared the distribution of ATAC-seq peaks to Roadmap Epigenomics Consortium chromatin states in adipose nuclei isolated from subcutaneous adipose tissue.(Roadmap Epigenomics Consortium 2015) We used the top 50,000 representative peaks in each group of samples. For all ATAC-seq profiles, the majority of peaks were located in adipose nuclei promoter and enhancer states, with fewer peaks located in regions associated with closed chromatin (heterochromatin, polycomb states; Table S3). Our adipose tissue peaks showed the strongest overlap (40% enhancer, 49% promoter, 89% combined) with adipose nuclei promoters and enhancers compared to all other ATAC-seq profiles (Figure 1C, Table S3). With the exception of ENCODE adipose tissue, enhancer coverage was consistently higher for adipose tissue and adipocyte profiles compared to preadipocyte and GM12878 lymphoblast profiles, whereas promoter coverage was similar between all samples (Figure 1C, Table S3). The ENCODE adipose tissue profile had more peak bases in regions near transcription start sites and fewer peak bases in distal regions compared to all other profiles (Table S4), which may reflect technical differences in sample processing.

Third, to characterize the epigenome distribution of ATAC-seq peaks across cell types, we determined the overlap of representative peaks from each ATAC-seq group with enhancer chromatin states from 98 Roadmap tissues and cell types including adipose nuclei.(Roadmap Epigenomics Consortium 2015) Adipose tissue and tissue-derived adipocyte peaks showed the most overlap with adipose nuclei enhancers, and SGBS adipocytes showed the 4^th^ most overlap with adipose nuclei enhancers compared to enhancers in other tissue and cell types (Table S3). SGBS preadipocytes showed the most overlap with enhancers in fibroblast cell types, and adipose nuclei ranked 24^th^ among all cell types. As expected, GM12878 lymphoblast peaks showed much less overlap with adipose nuclei enhancers, consistent with the cell type-specific nature of enhancers.(Roadmap Epigenomics Consortium 2015) Across the three methods, our adipose tissue and SGBS ATAC-seq profiles showed strong similarity with existing adipocyte ATAC-seq profiles and with active regulatory element chromatin states in adipose nuclei.

### Cardiometabolic GWAS loci in ATAC-seq peaks

**Figure 2:**
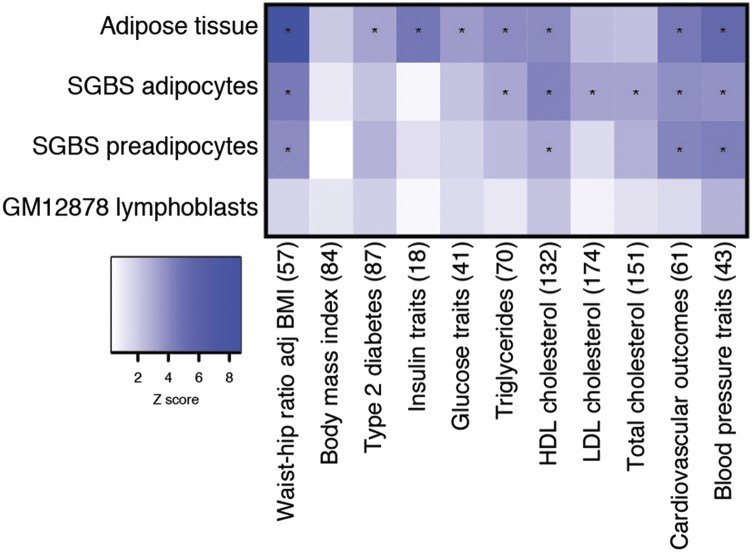
Cardiometabolic GWAS loci are enriched in ATAC-seq peaks. The heatmap shows enrichment of cardiometabolic GWAS loci (z-score) for the top 50,000 representative ATAC-seq peaks in adipose tissue, SGBS adipocytes, SGBS preadipocytes, and GM12878 lymphoblasts. Cells with a significant p-value (*P* < 0.005) contain an asterisk.

To identify cardiometabolic traits that may be strongly affected by adipocyte regulatory elements, we tested for enrichment of GWAS variants for 11 cardiometabolic trait groups (Table S5) in the top 50,000 representative ATAC-seq peaks in adipose tissue, SGBS adipocytes, SGBS preadipocytes, and GM12878 lymphoblasts. Variants at loci for four trait groups (WHR, HDL-C, cardiovascular outcomes, and blood pressure traits) showed significant enrichment (*P* < 5×10^−3^) in adipose tissue, SGBS adipocyte, and SGBS preadipocyte peaks (Figure 2, Table S6). WHR was the most strongly enriched trait in adipose tissue (z-score = 8.66) and SGBS adipocyte (z-score = 4.53) peaks, whereas blood pressure traits were most strongly enriched in SGBS preadipocyte peaks (z-score = 4.29). Loci for insulin traits and WHR showed stronger enrichment in adipose tissue peaks compared to SGBS adipocyte or preadipocyte peaks, suggesting *in vivo* conditions and/or non-adipocyte cell types in adipose tissue may contribute to these traits. Loci for HDL-C, triglycerides, LDL-C, and total cholesterol were significantly enriched in SGBS adipocytes, consistent with the roles of adipocytes in lipid storage. In contrast, loci for none of the tested traits were enriched in GM12878 lymphoblast peaks. Our results suggest that genetic variation in adipose tissue and adipocyte accessible chromatin regions is frequently associated with several cardiometabolic traits and that the stronger enrichment of WHR and insulin trait loci in adipose tissue relative to adipocyte or preadipocyte peaks demonstrates the importance of profiling chromatin accessibility in tissue.

### Functional evaluation of cardiometabolic GWAS variants overlapping ATAC-seq peaks

We next identified cardiometabolic GWAS variants that overlapped candidate regulatory elements defined by ATAC-seq peaks. We focused on ATAC-seq peaks at a subset of 110 cardiometabolic GWAS loci that were colocalized with gene expression quantitative trait loci (eQTLs) in subcutaneous adipose tissue;(Cannon *et al.* 2017; Civelek *et al.* 2017) these loci consisted of 6,692 variants (LD *r^2^* > 0.8 with lead GWAS variants). To strengthen annotation at these loci, we overlapped variants at these loci with all representative ATAC-seq peaks rather than the top 50,000 peaks. 147 variants at 59 loci overlapped an adipose tissue peak (Table S7). The loci that had only one variant overlapping an adipose tissue ATAC-seq peak are shown in Table 2; these variants are strong candidates for functional activity at these loci. Of these 147 variants, 136 (93%) also overlapped an SGBS adipocyte peak and 116 (79%) overlapped both an SGBS adipocyte and preadipocyte peak. Variants that overlap peaks in adipose tissue and adipocytes or preadipocytes may be more likely to act through regulatory elements present in adipocytes rather than blood, immune, or other adipose tissue cell type regulatory elements. Of the 147 variants, 97 (66%) overlapped a transcription factor (TF) motif from JASPAR.(Mathelier *et al.* 2016) Using a stringent definition for transcription factor footprints (Methods), we identified aggregate footprint profiles for 12 of 35 tested TF motifs in adipose tissue (Figures S1-S3, Table S8) and found that four variants overlapped a TF footprint. These candidate functional variants, target regulatory elements, and TFs provide a resource to investigate the mechanisms underlying cardiometabolic GWAS loci.

**Table 2:** Selected variants at GWAS-eQTL colocalized loci that overlap ATAC-seq peaks

GWAS trait	GWAS locus	GWAS index variant	Colocalized eQTL gene(s)	eQTL index variant(s)	Variant in ATAC-seq peak	Total variants (*r^2^* > 0.8) at locus	ATAC samples
Adiponectin	*GNL3*	rs2590838	*GNL3*, *NEK4*	rs35212380, rs7612511	rs1108842	21	1, 2, 3, Adipocytes, Preadipocytes
Coronary heart disease	*LIPA*	rs1412444	*LIPA*	rs1412445	rs1332328	8	3, Adipocytes, Preadipocytes
HDL cholesterol	*GSK3B*	rs6805251	*GSK3B*	rs334533	rs334558	61	1, 2, 3, Adipocytes, Preadipocytes
Intracranial aneurysm	*STARD13*	rs9315204	*KL*, *STARD13*	rs1998728, rs614691	rs1980781	22	1, 2, 3, Adipocytes, Preadipocytes
Serum metabolites	*NAT8*	rs13391552	*ALMS1*	rs6740766	rs4547554	180	1, 2, 3, Adipocytes
Proinsulin	*MADD*	rs10501320	*ACP2*, *FNBP4*	rs10501320, rs11039149	rs11039149	7	1, 2, 3, Adipocytes, Preadipocytes
Total cholesterol	*DOCK7-ANGPTL3*	rs2131925	*DOCK7*	rs631106	rs631106	237	1, Adipocytes, Preadipocytes
Triglycerides	*FADS1*	rs174548	*FADS1*	rs174555	rs174561	48	1, 2, 3, Adipocytes, Preadipocytes
Type 2 diabetes	*MPHOSPH9*	rs1727313	*C12orf65*, *CDK2AP1*, *SBNO1*	rs11057206, rs1616131, rs28583837	rs7485502	215	1, Adipocytes, Preadipocytes
WHRadjBMI	*SNX10*	rs1534696	*CBX3*, *SNX10*	rs1534696	rs1534696	1	1

A subset of loci in which only one variant overlapped an ATAC-seq peak at a colocalized GWAS-eQTL locus in adipose tissue,^9^ SGBS preadipocytes and/or SGBS adipocytes; all variants are listed in Table S7.

**Figure 3:**
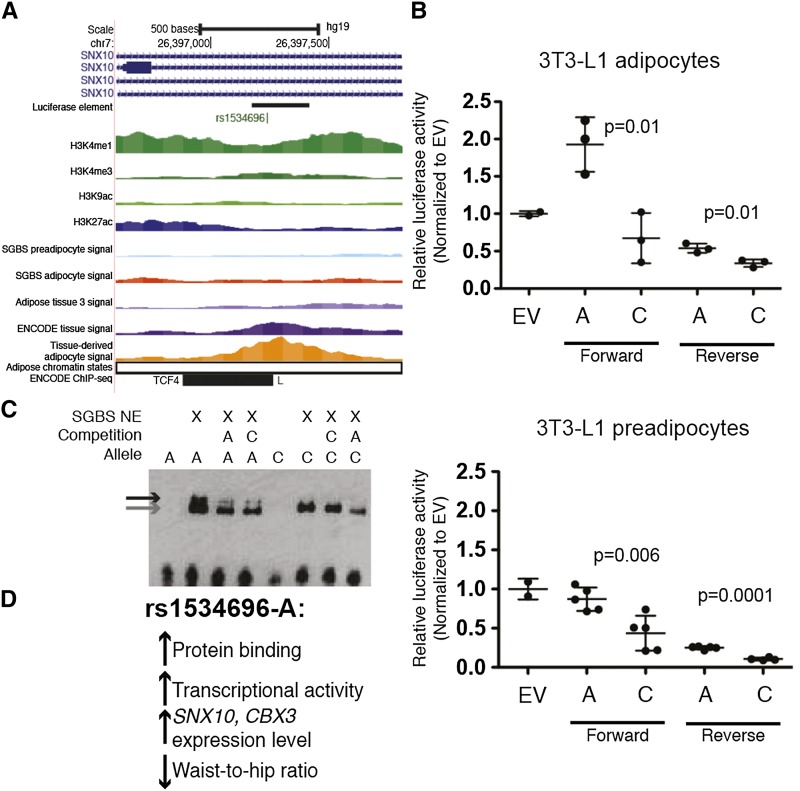
A variant at the *SNX10* WHR GWAS locus alters transcriptional activity and protein binding. (A) rs1534696 overlaps an ATAC-seq peak (adipose tissue 3 is shown in the figure; adipose tissue 1 shows stronger signal and peak) and is located in intron 2 of *SNX10*, transcribed left-to-right in the image, but is not located in a predicted regulatory region based on Roadmap chromatin states. TCF4 ENCODE ChIP-seq binding was observed in HepG2 cells. (B) The genomic region containing rs1534696-A shows increased transcriptional activity and allelic differences in transcriptional reporter luciferase assays in 3T3-L1 adipocytes and preadipocytes. The genomic region was cloned upstream of a minimal promoter and the luciferase gene. Dots represent the average of 2-3 technical replicates. Forward and reverse were designated with respect to the genome, so forward corresponds to left-to-right in the image. P-values determined by Student’s *t*-test. EV, empty vector. (C) rs1534696-A shows increased protein binding in EMSA using SGBS preadipocyte nuclear extract. The black arrow shows allelic differences in protein binding. The gray arrow denotes non-specific binding observed for both rs1534696-A and rs1534696-G. (D) Summary of the direction of effect of rs1534696-A. Additional regulatory assays are shown in Figure S4.

We tested variants at two loci for allelic differences in functional regulatory assays. The first, rs1534696, was identified as a candidate regulatory variant based on overlap with an ATAC-seq peak in adipose tissue and tissue-derived adipocytes, but was not a candidate based on SGBS adipocyte or preadipocyte ATAC-seq peaks or adipose promoter or enhancer Roadmap chromatin state (Figure 3A). rs1534696 is located in the second intron of *SNX10* (encoding sorting nexin 10), was associated with WHR (*P =* 2×10^−8^, β=0.027, in women)(Shungin *et al.* 2015) and exhibited a colocalized eQTL for *SNX10* (*P =* 3.4×10^−150^, β=1.12) and *CBX3* (*P =* 1.1×10^−13^, β=0.39) in adipose tissue.(Civelek *et al.* 2017) We tested alleles of rs1534696 in a 250-bp region encompassing the ATAC-seq peak for transcriptional differences in luciferase reporter assays using four cell types (Figure 3B, Figure S4). In 3T3-L1 preadipocytes and adipocytes, the construct containing rs1534696-A showed higher transcriptional activity than rs1534696-C (*P* = 0.01) in both orientations (Figure 3B). Similar trends were also observed in SW872 liposarcoma and SGBS preadipocyte cells (Figure S4); this direction of effect is consistent with the eQTL association of rs1534696-A with higher levels of *SNX10* and *CBX3*. In addition, rs1534696-A showed increased protein binding in EMSAs using nuclear extract from SGBS preadipocytes (Figure 3C). These data suggest that a transcriptional activator binds more strongly to rs1534696-A and increases transcriptional activity of *SNX10* and/or *CBX3*, contributing to the molecular mechanism at this GWAS locus (Figure 3D).

**Figure 4:**
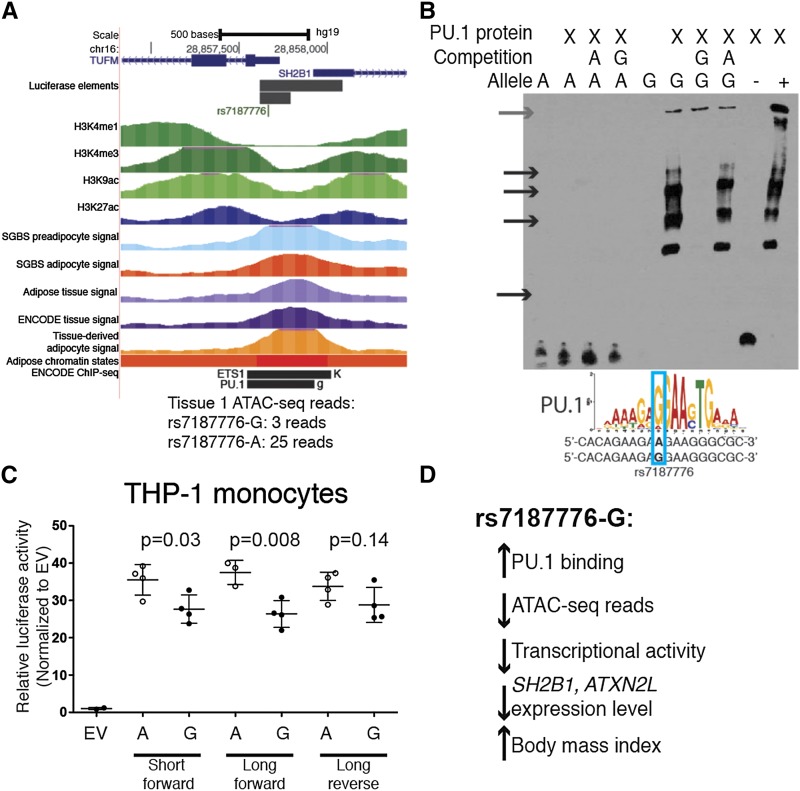
A variant at the *ATP2A1-SH2B1* BMI GWAS locus alters chromatin accessibility and PU.1 binding. **(**A) rs7187776 is located in the promoter of a long *SH2B1* isoform, transcribed left-to-right in the image; the 5′-UTR of *TUFM*, transcribed right-to-left in the image; and a region containing ATAC-seq peaks from multiple sources. ETS1 and PU.1 ENCODE ChIP-seq binding was observed in K562 and GM12891, respectively. Many additional transcription factor ChIP-seq peaks overlap this region in the ENCODE datasets. (B) A 19-nt probe containing rs7187776-G shows increased protein binding to purified PU.1 in EMSA, similar to a positive control probe containing the consensus PU.1 motif (+). A negative control probe (-) and a probe containing rs7187776-A showed no binding to PU.1. Black arrows indicate allele-specific protein binding, gray arrow indicates the well of the gel. Similar protein binding patterns and equal amounts of free DNA probe were observed using SW872 nuclear extract (Figure S5). PU.1 consensus motif from JASPAR (Mathelier *et al.* 2016). (C) The genomic region containing rs7187776-A shows increased transcriptional activity and allelic differences in THP-1 monocytes. The genomic region including part of the 3′ UTR of *TUFM* and part of the 5′ UTR of *SH2B1* was cloned upstream of a minimal promoter and the luciferase gene. Dots represent the average of two technical replicates. Forward and reverse designated with respect to the genome, so forward corresponds to left-to-right in the image. P-values determined by Student’s *t*-test. EV, empty vector. (D) Summary of the direction of effect of rs7187776-G.

The second variant we tested overlapped an ATAC-seq peak in adipose tissue, SGBS preadipocytes, SGBS adipocytes, and tissue-derived adipocytes and a SPI1 (PU.1) ChIP-seq peak, motif and footprint (Figure 4A). In adipose tissue sample 1, we further observed an allelic imbalance in ATAC-seq reads (*P* = 2.90×10^−3^): 25 reads contained rs7187776-A and 3 reads contained rs7187776-G. rs7187776 is located near a long isoform of *SH2B1* (encoding SH2B adaptor protein 1) and is in strong LD (*r^2^* > 0.8) with the lead variant associated with BMI (rs3888190, *P =* 3.14×10^−23^, β=0.031).(Locke *et al.* 2015) This GWAS signal exhibited a colocalized eQTL for *SH2B1* (*P =* 4.7×10^−15^, β=-0.39) and *ATXN2L* (*P =* 2.5×10^−11^, β=-0.34) in adipose tissue.(Civelek *et al.* 2017) rs7187776 is one of 124 candidate variants based on LD (*r^2^* > 0.8) with the lead GWAS and eQTL variants, and one of five variants that overlapped ATAC-seq peaks at this locus (Table S7). Using EMSA, we observed allele-specific binding of rs7187776-G to purified PU.1 protein and similar binding using nuclear extract from SW872 cells, consistent with the predicted motif (Figure 4B, Figure S5). We also tested alleles of rs7187776 in a 477-bp region encompassing the ATAC-seq peak and a smaller 186-bp region in transcriptional reporter assays (Figure 4, Figure S5). In THP-1 monocytes, the constructs containing rs7187776-A showed increased transcriptional activity compared to rs7187776-G (Figure 4C). In SGBS preadipocyte, SW872 liposarcoma, 3T3-L1 preadipocyte, and 3T3L-1 adipocyte cells, we observed extremely strong transcriptional activity (>200-fold compared to background) but no allelic differences (Figure S5); differences may have been masked by the massive >200-fold transcription-enhancing effect of this region. rs7187776-G is associated with decreased expression levels of *SH2B1* and *ATXN2L*, suggesting that PU.1 or another ETS family member may act as a transcriptional repressor at this locus. We observed fewer ATAC-seq reads corresponding to more PU.1 binding, a direction that has been observed less often than increased ATAC-seq reads corresponding to increased transcription factor binding. (Degner *et al.* 2012) We observed the same pattern in GM12891 SPI1 ChIP-seq and DNase-seq data from ENCODE; 2 ChIP-seq reads contained rs7187776-A and 11 reads contained rs7187776-G, whereas 11 DNase-seq reads contained rs7187776-A and 1 read contained rs7187776-G. Multiple ETS family members, including PU.1, can act as transcriptional repressors, including by recruiting histone deacetylases and DNA methyltransferases, resulting in closed chromatin, (Suzuki *et al.* 2006; Suzuki *et al.* 2003; Kihara-Negishi *et al.* 2001; Yashiro *et al.* 2015) consistent with rs7187776-G showing fewer ATAC-seq reads. These data suggest that rs7187776-G increases binding of an ETS family member, and may contribute to the molecular mechanism at the *ATP2A1-SH2B1* BMI GWAS locus (Figure 4D).

### Allelic imbalance in ATAC-seq reads

We looked for other examples of allelic imbalance in ATAC-seq reads at heterozygous positions that may indicate altered chromatin accessibility. Only 387 sites showed nominal allelic imbalance (beta-binomial *P* < 0.05) in at least one sample (Table S9), 6 of which overlapped variants at GWAS-eQTL loci (Table S10). However, only 40 of 6,692 total GWAS-eQTL variants were heterozygous in at least one adipose tissue sample and were covered by enough ATAC-seq reads for allelic imbalance analysis, suggesting that higher read depth and larger sample sizes that increase the chance of heterozygosity at more eQTL and GWAS loci may enable identification of more disease-associated loci that could mediate their effects on disease through chromatin accessibility.

## Discussion

In this study, we generated ATAC-seq open chromatin profiles from three frozen clinical adipose samples and replicate preparations of SGBS preadipocytes and adipocytes. We identified differences between adipose tissue, preadipocyte, and mature adipocyte open chromatin profiles, including cell-type-specific peaks at selectively expressed promoters. Adipose tissue, SGBS adipocyte, and SGBS preadipocyte open chromatin profiles largely overlapped Roadmap adipose nuclei chromatin states. Transcription factor motifs and footprints in ATAC-seq peaks overlapped GWAS variants, and GWAS variants for several traits were enriched in ATAC-seq peaks. Finally, we used the ATAC-seq profiles to annotate potential regulatory variants at GWAS-eQTL colocalized loci and provided experimental evidence of allelic differences in regulatory activity for variants at the *SNX10* and *ATP2A1-SH2B1* GWAS loci. Taken together, these data are among the deepest characterization of chromatin accessibility in adipose tissue, adipocytes, and preadipocytes to date.

Important differences exist between adipose tissue, preadipocyte, and mature adipocyte ATAC-seq profiles. Explanations for these differences include cell-type composition/heterogeneity, the differentiation state of adipocytes, the cultured nature of SGBS cells, and technical differences of ATAC-seq data (*e.g.*, sequencing depth). At the TSS for *ADIPOQ*, we observed adipose tissue and SGBS adipocyte ATAC-seq peaks, and downstream of *ADIPOQ*, we observed ATAC-seq peaks specific to SGBS preadipocytes. The accessibility pattern of *ADIPOQ* is consistent with its role in adipocyte differentiation (Schäffler *et al.* 1999; Yamauchi *et al.* 2002; Yokota *et al.* 2002) and a previous finding that the *ADIPOQ* promoter is inaccessible until differentiation (Musri *et al.* 2006). Among 98 Roadmap tissue and cell types, SGBS preadipocyte ATAC-seq profiles were more similar to fibroblast-like cells and cell lines than to adipose nuclei, and SGBS adipocytes were more similar to adipose nuclei, reflecting differences likely due to the fibroblast-like nature of preadipocytes. Differences between our adipose tissue ATAC-seq profiles and the ENCODE adipose tissue data may be due to differences in biopsy location, freezing method, storage conditions, or library preparation.

Adipose ATAC-seq profiles provide insight into the mechanisms of cardiometabolic GWAS loci. For example, we found that GWAS variants for WHR— but not BMI—are enriched in adipose ATAC-seq peaks. This enrichment is consistent with recent findings that WHR loci are enriched in adipose transcriptional regulatory elements(Shungin *et al.* 2015) and that BMI GWAS loci are enriched in pathways involved in central nervous system biology.(Locke *et al.* 2015) We also identified enrichment of other cardiometabolic traits, including insulin traits, lipids, and cardiovascular outcomes, highlighting the relevance of adipose regulatory elements for these traits. Identifying the transcription factor(s) bound to a regulatory variant is a challenging part of defining the molecular mechanisms underlying cardiometabolic GWAS loci. While transcription factor footprints better predict that a transcription factor is bound at a locus compared to motif occurrence alone,(Pique-Regi *et al.* 2011) neither footprints nor motifs identify the bound transcription factor with 100% accuracy, particularly when multiple transcription factors share similar binding motifs. We successfully generated transcription factor footprints for 12 transcription factor motifs (Figures S1-S3), which can be used to identify GWAS variants that may alter transcription factor binding. However, additional experiments are needed to confirm the identity of transcription factors bound at loci containing these footprints.

We described two GWAS loci for which ATAC-seq peaks helped prioritize candidate variants. At the *SNX10* WHR locus, we identified a potentially functional variant, rs1534696, which is not located in a predicted regulatory region based on existing chromatin state data. rs1534696 overlaps an ATAC-seq peak in adipose tissue and showed allelic differences in transcriptional reporter and protein-binding assays. Interestingly, we observed allelic differences in protein binding in SGBS preadipocytes, yet low transcriptional activity, similar to empty vector, in SGBS preadipocytes and 3T3L1 cells. One possibility is that a repressor binds in preadipocytes to prevent transcription and is then released to activate transcription in adipocytes; additional experiments are needed to determine the apparent differences between preadipocytes and adipocytes at this locus. At the *ATP2A1-SH2B1* BMI locus, we identified a PU.1 binding motif and footprint at rs7187776, as well as allelic imbalance in ATAC-seq reads, and confirmed the allelic differences in PU.1 binding *in vitro*. PU.1 is part of the ETS family of transcription factors, all of which have very similar DNA binding motifs,(Wei *et al.* 2010) so PU.1 may not be the specific TF binding at this locus, especially because PU.1 is expressed at very low levels in SGBS preadipocytes, SGBS adipocytes, and isolated mature adipocytes.(Allum *et al.* 2015; Schmidt *et al.* 2015b) Interestingly, we observed significant allelic differences in transcriptional activity in THP-1 monocyte cells but not in preadipocyte or adipocyte cell types (Figure 4 and Figure S5), suggesting that this variant might be important in non-adipocyte cells within adipose tissue. These data provide excellent examples of how to integrate GWAS, eQTL, and ATAC-seq data to identify functional variants at GWAS loci. Further experiments are needed to determine if these variants are the only functional variants at each locus, as we also observed allelic differences in protein binding for a second variant overlapping an ATAC-seq peak at the *SH2B1* locus (Figure S5B) and others have suggested different functional variants at this locus,(Giuranna *et al.* 2018; Volckmar *et al.* 2012) and which gene(s) are contributing to obesity risk.

In summary, we presented ATAC-seq open chromatin profiles for frozen adipose tissue and cultured preadipocytes and adipocytes. We showed the utility of open chromatin profiles in multiple tissue samples and across cell types within heterogeneous tissue. Together, these data add to the growing understanding of gene regulation in adipose and the complex genetic mechanisms of cardiometabolic traits and diseases.
